# Biocompatibility of fluids for multiphase drops-in-drops microfluidics

**DOI:** 10.1007/s10544-016-0137-0

**Published:** 2016-12-05

**Authors:** Aishah Prastowo, Alexander Feuerborn, Peter R. Cook, Edmond J. Walsh

**Affiliations:** 1 Osney Thermo-Fluids Laboratory, Department of Engineering Science, University of Oxford, Osney Mead, Oxford, OX2 0ES UK; 2 Sir William Dunn School of Pathology, University of Oxford, South Parks Road, Oxford, OX1 3RE UK

**Keywords:** Biocompatibility, Drops-in-drops, Drug screening, Mammalian cell, Toxicity, Surfactant

## Abstract

This paper addresses the biocompatibility of fluids and surfactants in the context of microfluidics and more specifically in a drops-in-drops system for mammalian cell based drug screening. In the drops-in-drops approach, three immiscible fluids are used to manipulate the flow of aqueous microliter-sized drops; it enables merging of drops containing cells with drops containing drugs within a Teflon tube. Preliminary tests showed that a commonly-used fluid and surfactant combination resulted in significant variability in gene expression levels in Jurkat cells after exposure to a drug for four hours. This result led to further investigations of potential fluid and surfactant combinations that can be used in microfluidic systems for medium to long-term drug screening. Results herein identify a fluid combination, HFE-7500 and 5-cSt silicone oil + 0.25% Abil EM180, which enabled the drops-in-drops approach; this combination also allowed gene expression at normal levels comparable with the conventional drug screening in both magnitude and variability.

## Introduction

Drop-based microfluidics is expected to play an important role in drug discovery (Dittrich and Manz 2006; Dressler et al. 2014; Kang et al. 2008; Tsui et al. 2013) through increased efficiency coupled to large-scale parallelization (Gong et al. 2011; Miller et al. 2012); then many compounds in many different concentrations (Churski et al. 2012; Hong et al. 2016) can be screened in different cell types (Gao et al. 2013; Yu et al. 2009). It can also facilitate single-cell analyses (Rodriguez-Rodriguez et al. 2012). In all these cases, there would be a simultaneous reduction in volumes and cost. However, current microfluidic systems suffer from various drawbacks that are limiting wide acceptance (Sackmann et al. 2014); for example, they often contain complicated network of channels that are difficult to fabricate (Friend and Yeo 2010; Mazutis et al. 2013), they require sophisticated additional machinery (Hansen et al. 2015; Kellogg et al. 2014), and one chip design is usually limited to one specific application (Fiorini and Chiu 2005; Friend and Yeo 2010).

**Fig. 1:**
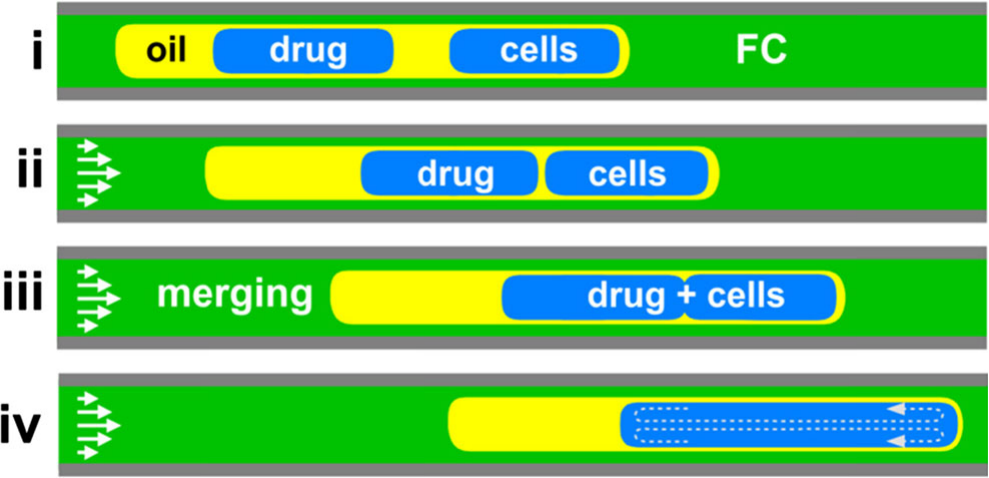
Merging drops-in-drops in a Teflon tube. (i) Initial structure of two aqueous drops – which contain cells or a drug – engulfed in one oil super-drop, which is engulfed in turn in a fluorocarbon (FC). This structure spontaneously forms as the end of the tube (which is connected to a syringe pump acting in withdrawal mode, and which is pre-filled with fluorocarbon) is dipped successively into fluorocarbon, oil, growth medium containing cells, oil, growth medium containing the drug, and fluorocarbon. (ii) As a result of the oil film surrounding the aqueous drops and the parabolic laminar-flow profile, the engulfed aqueous drops containing cells moves faster than the oil. When the right-hand drop reaches the leading interface of the oil, it slows to travel at the velocity of the oil. With continued flow, the trailing left-hand drop containing the drug eventually catches up the leading one containing cells. (iii) Once the two aqueous drops touch, they merge. (iv) Internal vortices within the merged drop mix contents. This Figure was adapted from (Feuerborn et al. 2015)

An alternative microfluidic method utilising a Teflon tube and fluid mechanics, rather than the more common approach of relying on the geometry of micro-scale channel networks, has recently been developed (Feuerborn et al. 2015; Walsh et al. 2016). This method exploits the interfacial tension between three or more immiscible liquids to create specific fluidic architectures. In this context, we consider the particular architecture of two aqueous drops engulfed within one oil drop, which is – in turn – surrounded by a fluorocarbon. As a result of the liquid films surrounding drops at different points within the system, the relative velocities of the two aqueous drops can be controlled; the two drops can be forced to merge, and their contents mixed (Fig. 1).

To create the required fluidic architecture in the three-phase system, fluids must have the appropriate interfacial tensions. This can be achieved when the interfacial tension between the fluorocarbon (FC) and the aqueous phase (γ_FC/aq_) is greater than the sum of the interfacial tension between fluorocarbon and oil (γ_FC/oil_) plus that between oil and water (γ_oil/aq_). In other words, γ_FC/aq_ > γ_FC/oil_ + γ_oil/aq_, as defined by the Neumann triangle (Chen et al. 2007; Guzowski et al. 2012). This fluidic architecture, and the mechanism that drives the merging of the two aqueous drops, are illustrated in Fig. 1. The time needed for the second drop to catch up the first one depends on the initial spacing between drops (established when the two drops first enter the tube) and their relative motion thereafter (Feuerborn et al. 2015). This relative velocity is influenced by the thickness of the fluidic film surrounding the drops, which varies with Capillary number (Ca *= ηU/γ*, where *η* = carrier fluid viscosity and *U* = average velocity) (Bico and Quéré 2000; Bretherton 1961).

Many fluid/surfactant combinations can be found that satisfy the Neumann triangle, and allow drops-in-drops to form and merge. However, for cell-based assays, these fluid/surfactant combinations must also be biocompatible. Surfactants are widely used in many other microfluidic drop-based systems used for biological applications, and – and as many fluid/surfactant combinations are toxic to cells (Baret 2012; Pang et al. 2006; Partearroyo et al. 1990) – biocompatibility of the liquids used is a general problem. The definition of a biocompatible environment varies within the microfluidic literature, and few studies have focused on this in a rigorous way. One measure used to claim biocompatibility is the ability to grow cells after they have been through a microfluidic device (Huang et al. 2015; Liu et al. 2009; Martin et al. 2003). However, most cells respond to a toxic environment by arresting growth, and then they may be able to “recover” from the sub-optimal environment when returned to a favourable one. Consequently, cell behaviour may differ inside and outside the device. In addition, several authors claim biocompatibility by citing a previous study using similar fluids. For example, several articles cite ref. (Clausell-Tormos et al. 2008) to support proliferation within drops; however, the original authors (who counted the ratio of living and dead cells) only found “some degree of proliferation within the drops”. Here, we define a biocompatible environment as one in which the gene expression levels of cells in drops are comparable to those found using the same cells growing in a conventional tissue-culture flask. This definition has particular relevance in the case of a cell grown in suspension like the Jurkat cell – an immortalized line of human T lymphocytes – where assessment of cell morphology is more difficult than it is with adherent cells.

Here we initially utilised the fluid/surfactant concentrations employed by others for drop-based microfluidics using cells (i.e., HFE-7500 and tetradecane + Span 80) (El Debs et al. 2012; Gu et al. 2011; Hu et al. 2015; Li et al. 2014; Martin et al. 2003; Schoeman et al. 2014) with our new approach. We used a low concentration of surfactant (i.e., 0.25% *w*/w Span 80) to minimise potential toxicity. To verify biocompatibility, we first measured levels of 5S ribosomal RNA (rRNA) in Jurkat cells, as this is commonly used as a control (Tea et al. 2013). Levels (quantified using qRT-PCR) in cells from different drops were found to vary sporadically. This led us to examine the reason for this sporadic behaviour, and this was traced to the fluids/surfactants employed. We then went on to screen many fluid/surfactant combinations to see which affected cell viability and gene expression. We found that many commonly-used combinations had negative effects on cells over periods of a few hours. We also identified a fluid/surfactant combination (i.e., HFE-7500 as carrier fluid, 5-cSt silicone oil +0.25% Abil EM180 as separating fluid, and cell-culture media as the aqueous fluid containing cells and/or drugs) that could be used in our system and which was biocompatible (assessed by comparing viability and expression levels using cells grown conventionally in micro-wells).

## Experimental methods

### Interfacial tension measurement

The interfacial tension between two immiscible fluids was measured using a commercial instrument (First Ten Angstroms) employing the pendant-drop method. Fluid with higher density was loaded in a syringe, a small drop was formed at the tip of the needle, and the drop was immersed in the second fluid contained in a transparent polystyrene cuvette. The interfacial tension was calculated using the manufacturer’s software, a length scale (i.e., the width of the needle tip measured with a micrometre), and fluid density.

### Cell preparation

Jurkat or EL4 (mouse lymphoblast) cells were cultured routinely in flasks in RPMI-1640 supplemented with 10% fetal bovine serum and 1% penicillin and streptomycin. They were used at 500 cells/μl for cell-viability assays (allowing cells to grow for up to 48 h), or 2000 cells/μl for drug-screening tests (for 4 h tests).

### Fluid and surfactant biocompatibility test

Different biocompatibility tests with different surface-to-volume ratios between aqueous drop and separating fluid were undertaken by overlaying the aqueous layer containing cells with fluid in a 96-well plate (Fig. 3 a(i)) and using a Teflon tube containing aqueous drops in two- or three-phase systems (Fig. 3 b(i), Fig. 3 c(i)).

The initial screen involved 150 μl separating fluid and cells in the same well of a 96-well plate (Fig. 3 a(i)); the non-adherant cells sediment under gravity to sit on the bottom of the well, or – if the separating fluid is the densest – on the interface between the two liquids. After incubating the plate at 37°C in 5% CO_2_, the percentage of live cells was determined using trypan-blue exclusion and a hemocytometer after mixing equal volumes of cell solution and 0.4% trypan blue.

The fluids that had no effect on the viability in the initial screen were tested (Fig. 3 b(i)) by withdrawing drops into a Teflon tube (bore 560 μm) connected to a syringe pump (Harvard Ultra). The tube was filled with test fluid, 1 μl drops of cell solution were withdrawn into the tube at a flow rate of 2–5 ml/h, and the tube sealed at both ends and incubated at 37°C in 5% CO_2_ for up to 48 h. Viability was assessed as before using trypan-blue exclusion by ejecting fluid from the tube at a flow rate of 2 ml/h until 10 drops were deposited into a drop of equal volume of trypan blue.

Biocompatibility was next tested using a static three-phase system in a Teflon tube containing the fluidic architecture to be used in a drug screen (Fig. 3 c(i)). In this screening the selected separating fluids are mixed with surfactant: Span 80 (sorbitan monooleate), Abil EM90, and Abil EM180 (Cetyl PEG/PPG-10/1 Dimethicone). A tube filled with fluorocarbon HFE-7500 was dipped successively into fluids contained in different wells in a 96-well plate; using a flow rate of 2–5 ml/h, the tube was dipped successively into cells (2 μl), separating fluid ± surfactant (1 μl), and HFE-7500 (5 μl). Finally, cells were incubated and viability was assessed as above.

The final biocompatibility test used conditions replicating those found in a drug screen – which involves flow down the tube to drive the merging of drops and delivery of drug to cells. First, the tube was filled with HFE-7500, and – as fluid was withdrawn into the tube – the end was dipped successively into HFE-7500 (to load 5 μl), cells in medium (to load a 1 μl drop), separating fluid (to load 200 μl), cells in medium (another 1 μl drop), and then HFE-7500 (to load 5 μl). This creates a “train” of aqueous drops; repeating this process generates further trains separated by 5 μl carrier fluid.

To test the effect of fluid and surfactant on gene expression, a drop containing Jurkat cells and one containing 0.5% (*v*/v) dimethyl sulfoxide (DMSO) were merged (at least 10 pairs in one tube). The carrier fluid used was HFE-7500, and the separating oil was tetradecane + 0.25% Span80 or 5cSt silicone oil + 0.25% Abil EM180. Five drops were ejected from the tube immediately after loading and each was put into separate Eppendorf tubes containing 12 μl “CellsDirect resuspension and lysis buffer” (CellsDirect™; Life Technologies). The remaining drops in the tube were incubated at 37°C, 5% CO_2_ for 4 h before being ejected individually into lysis buffer. This time was chosen because cells respond to one of the drugs used over this period (Diehn et al. 2002). As a control, 100 μl cells were plated in a well in a 96-well plate. 2 μl samples were taken from the plate after incubation for 0 and 4 h and mixed with lysis buffer following the same procedure used with samples from the drops-in-drops. Samples from both drops-in-drops and the control were lysed for 10 min at 75°C. The level of 5S rRNA in each sample was assessed using qRT-PCR (PCR cycles were 50°C for 20 min, 95°C for 5 min, and 40 cycles at 95°C for 15 s + 60°C for 30 s).

### Proof-of-principle drug screening

To demonstrate the inhibition and activation of gene expression in Jurkat cells, the following drugs were used: 100 μM 5, 6-dichloro-1-β-D-ribofuranosyl-benzimidazole (DRB) as inhibitor, and 1 μg/ml ionomycin + 62.5 ng/ml phorbol 12-myristate 13-acetate (PMA) as activator. Jurkat cells and drugs or 0.5% (v/v) DMSO (as control) were taken into the tube using the same method as the previous test. The carrier fluid was HFE-7500 and the separating oil was 5-cSt silicone oil + 0.25% Abil EM180. After incubation for 4 h, each sample was ejected into 12 μl lysis buffer, and treated as for the previous test. Levels of c-MYC mRNA and IL2 mRNA were also assessed using the ΔΔCt qRT-PCR method (Livak and Schmittgen 2001) and RT-PCR plus gel electrophoresis, respectively.

## Results and discussion

### Effects of tetradecane plus Span 80 on viability

HFE-7500 plus tetradecane/Span 80 plus growth medium had surface tensions satisfying the requirements of the Neumann triangle. When 0.25% Span 80 (weight/weight) – an oil-soluble surfactant that significantly reduces the oil-media interfacial tension from 17.7 to ~1.9 mN/m) – is added to tetradecane, it has little effect on the interfacial tension between HFE-7500 and tetradecane. As this combination had a particularly suitable alignment of interfacial tensions for use in our assay, and the fluids had previously been used for applications in biology (El Debs et al. 2012; Gu et al. 2011; Hu et al. 2015; Li et al. 2014; Martin et al. 2003; Schoeman et al. 2014), we explored its biocompatibility.

**Fig. 2:**
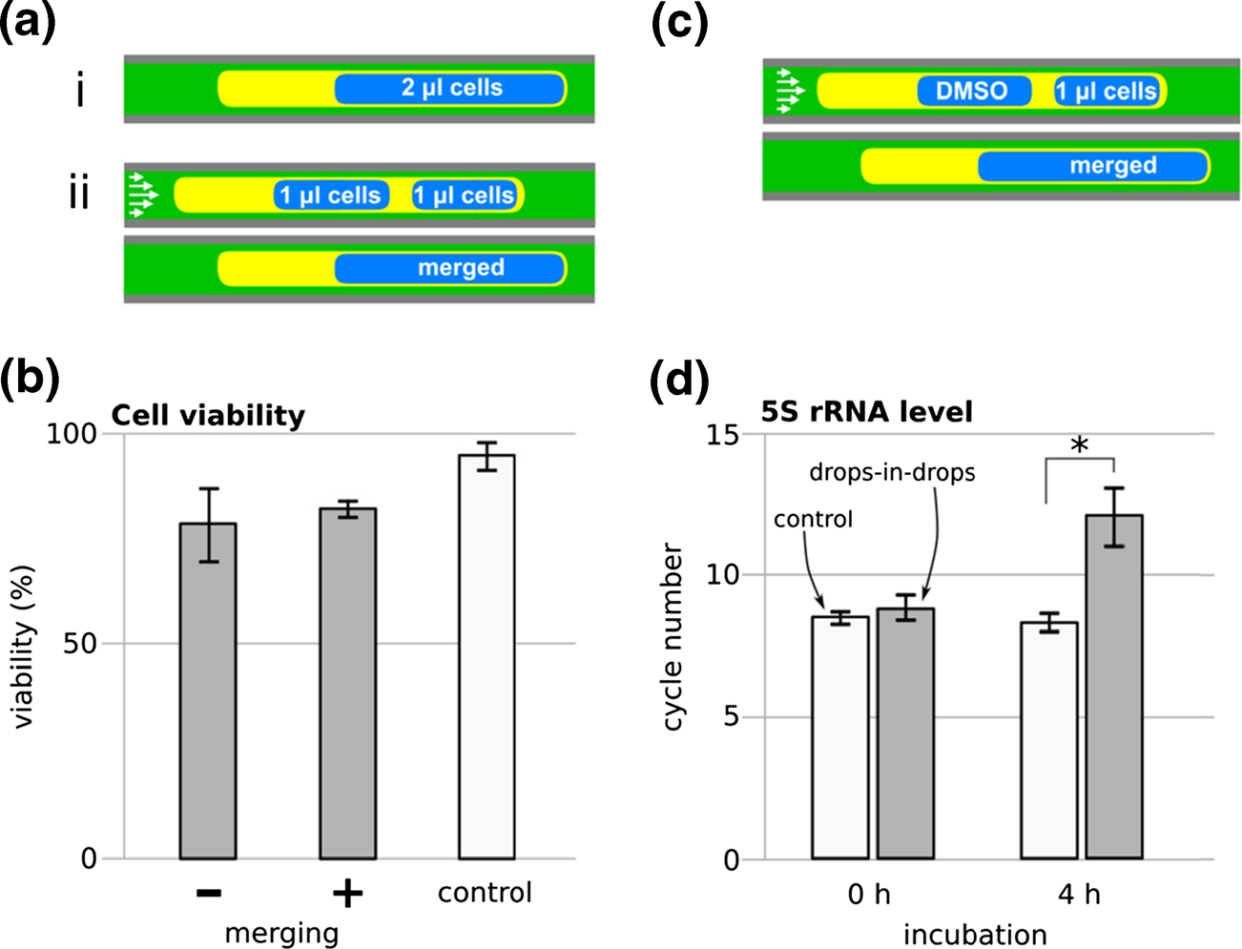
Effects of tetradecane and Span 80 on cell viability and levels of 5S rRNA (Jurkat cells). (**a**) Two approaches used to assess viability: (i) no merging, static (2-μl drops in separating oil), and (ii) merging, dynamic (two 1-μl drops were loaded, and drops merged). (**b**) Viability (defined as the percentage of live cells in the population assessed using trypan-blue exclusion after ‘no merging’ or ‘merging’ followed by a 4-h incubation. The three phases were growth medium, tetradecane + 0.25% Span 80, and HFE-7500. ‘Control’: viability after 4 h for the same cells grown conventionally in a 96-well plate. (**c**) Approach used for gene expression analysis. A drop containing cells was merged with another containing DMSO (the drug carrier). (**d**) Levels of 5S rRNA (assessed using qRT-PCR; a low cycle number reflects high levels of 5S rRNA). Cells were either taken into tubes and ejected immediately (‘0 h’) or incubated in tubes for 4 h. ‘Drops-in-drops’: cells in the dynamic three-phase system (HFE-7500, growth medium, and either tetradecane + 0.25% Span 80). ‘Control’: the same cells grown conventionally in 96-well plate. Error bars: ± standard deviation, *: significantly different (two-sample t-test, *p* < 0.05; *n* (control) = 3, *n* (drops-in-drops) = 7)

In our three-phase system, the carrier fluid HFE-7500 engulfs tetradecane and does not contact cells directly; therefore, the main fluids that might affect cell viability are tetradecane and Span 80, and their effects were investigated in two steps. First, as a quick screen, we incubated cells under a layer of tetradecane (with and without Span 80) in a 96-well plate. Viability (assess using trypan-blue exclusion) was >90% on exposure for 6 h to tetradecane, either on its own or with 0.25% or 1% Span 80. We then examined effects using the drops-in-drops structure in a tube, with and without drop merging. 0.25% Span 80 was used as the minimum amount of surfactant in tetradecane that facilitated a reliable merging. There was no significant impact of merging on cell viability (Fig. 2 b) using a level of 80% as a cut-off – a level that has been used in previous studies (Du et al. 2013; Qu et al. 2012; Sgro et al. 2007).

We then assessed levels of 5S rRNA in cells in drops-in-drops before and after 4 h incubation in the tube (using qRT-PCR). We first tested if loading and then immediately ejecting drops out of the tube affected cell number (and so 5S rRNA levels); it did not (in Fig. 2 d ‘0 h’, cycle numbers for ‘control’ and ‘drops-in-drops’ are comparable). However, after incubation in the drops for 4 h, levels fell (in Fig. 2 d ‘4 h’, more PCR cycles are required for detection). This result points to a deterioration in cell function. Therefore, we screened other fluids and surfactants for their potential use in our approach.

### Screening fluids for effects on viability

To explore alternative fluid/surfactant combinations that might be used to merge drops, we next listed the fluids/surfactants that had been used previously with mammalian cells in droplet-based microfluidics (Table 1). In our three-phase system, one fluid must be cell-growth medium, and the second is likely to be either HFE-7500 or FC-40 (i.e., the two fluorocarbons used most frequently to carry aqueous drops in microfluidic systems). Here, we note that HFE-7500 stably maintains the desired architecture of drops-in-drops better than FC-40. The third fluid has to be immiscible with fluorocarbon and water – and so probably a hydrocarbon, silicone oil, or vegetable oil. Addition of surfactants to this third fluid allows interfacial tensions to be tuned to satisfy the requirements of the Neumann triangle. However, surfactants are usually toxic to cells, and so the challenge is finding an appropriate combination of biocompatible fluids and surfactants.

**Table 1:** Fluids and surfactants previously used in drop-based microfluidics with mammalian cells

Fluid	Surfactant	Cell type
Fluorinated fluids		
FC-40	PEG-PFPE block copolymer	2C6 hybridoma (Koster et al. 2008), human U937 (Joensson et al. 2009), Chinese Hamster Ovary (Chen et al. 2011), human PC3, Raji B lymphocytes (Eastburn et al. 2013), HL60 (Edd et al. 2008), HEK293T (Juul et al. 2011), K562, U87 (Mongersun et al. 2016)
	DMP-PFPE block copolymer	HEK293T, Jurkat (Clausell-Tormos et al. 2008), Red blood cells (Abbyad et al. 2011; Abbyad et al. 2010)
HFE-7500	PEG-PFPE block copolymer	MDA-MB-231, PC9 (Ng et al. 2016), Her2 hybridoma (Hu et al. 2015), RAW 264.7 (Fischer et al. 2015), mouse ES (Klein et al. 2015), K-562 (Klein et al. 2015; Ng et al. 2016)
FC-3283	Perfluorooctanol (PFO)	Red blood cells (Kline et al. 2008), human periosteal cells (Srisa-Art et al. 2009)
Hydrocarbon/silicone/vegetable oils		
Hexadecane	Span 80	Chinese Hamster Ovary (Zhan et al. 2009), mouse myeloma cells (Kemna et al. 2013)
Tetradecane	Span 80	PC12 (Gu et al. 2011), HeLa, CCRF-CEM, Ramos (Li et al. 2014)
Mineral oil	Abil EM90	MDCK (Mary et al. 2011)
	Span 80	Jurkat, red blood cells (Chabert and Viovy 2008), Chinese Hamster Ovary (Hufnagel et al. 2009), leukemia cells (Sun et al. 2011), PC3 (Konry et al. 2011), human breast cancer cells
	Abil EM90 + Span 80	PC9 (Jing et al. 2015)
Silicone oil	-	HeLa (Xiao et al. 2010)
	Span 80	Mouse B lymphocytes (Sgro et al. 2007)
Soybean oil	-	Mouse mast cells and B lymphocytes (He et al. 2005)
Oleic acid	-	HeLa (Tan et al. 2006)

**Fig. 3:**
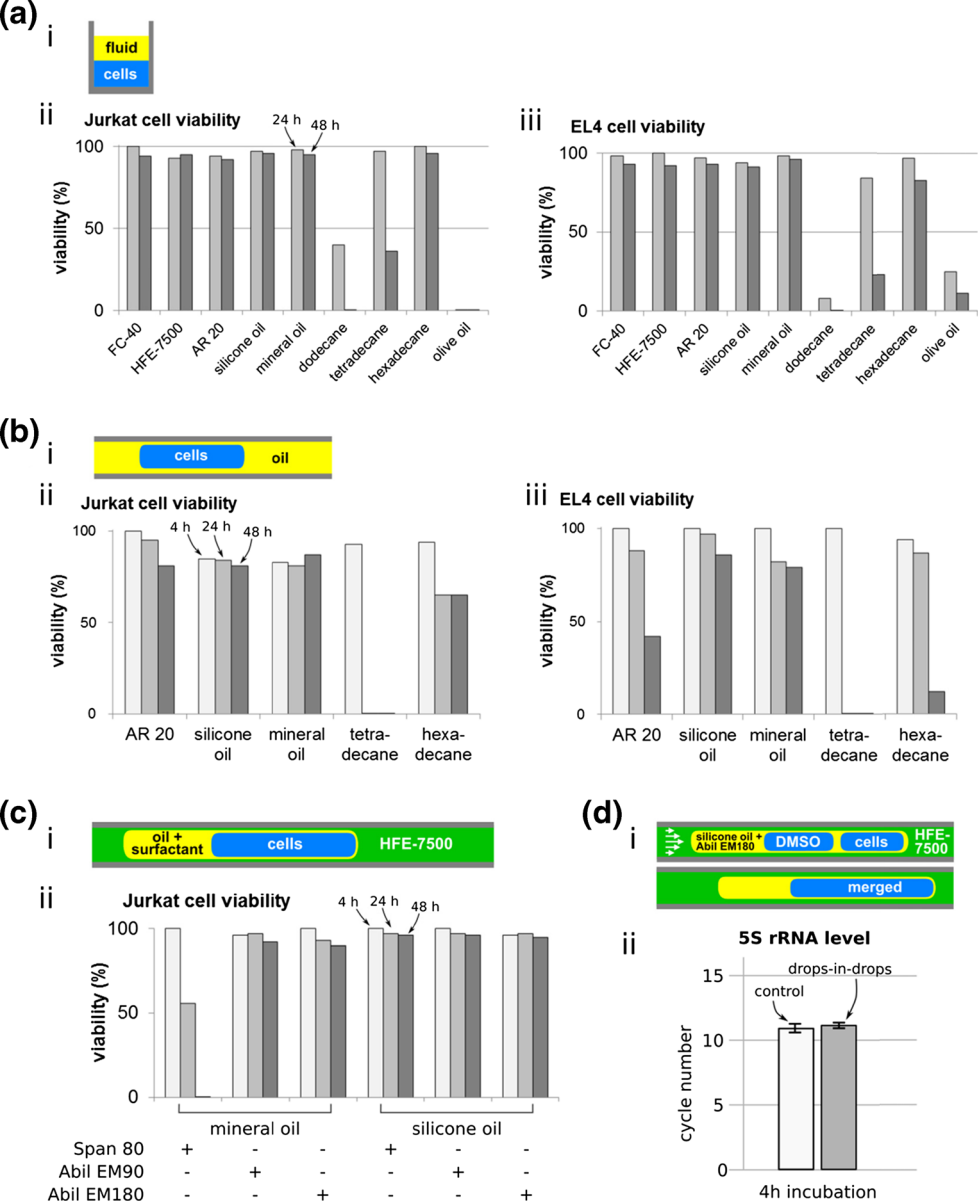
Effects of different fluids on viability of Jurkats and EL4. (**a**) Assay using an over- or under-lay in a 96-well plate (150 μl test fluid + 150 μl cells). (i) Cartoon illustrating approach (layers inverted, depending on density). (ii-iii) Viability after incubation with different fluids. Dodecane and olive oil gave poor viability, and so were not used subsequently. (**b**) Assay using 1-μl drops in a two-phase system in a tube. (i) Cartoon illustrating approach. (ii–iii) Viability after incubation with different fluids. Mineral oil and silicone oil AR 20 advanced to the next screening round. (**c**) Assay using 3-phase system. (i-ii) Viability of Jurkats assessed at different times using a static 3-phase system in a tube. 2 μl aqueous drops were engulfed in the separating oil indicated, which – in turn – was engulfed in HFE-7500. (**d**) Gene expression test using selected fluid/surfactant combination. (i) Cartoon illustrating approach (HFE-7500 as carrier, 5-cSt silicone oil + 0.25% Abil EM180 as separating oil). (ii) 5S rRNA levels (assessed using qRT PCR; a low cycle number reflects a high level). After 4-h incubation, levels in cells in drops-in-drops are comparable to those in cells grown conventionally

To find which combinations might be appropriate, biocompatibility was assessed in several steps. First, two fluorocarbons and seven separating oil candidates without surfactant were screened using Jurkats and EL4 cells in a 96-well plate. Viability was quantified after incubation for 24 and 48 h (Fig. 3 a). As dodecane and olive oil gave less than 50% viability after 24 h (with a further reduction after 48 h), they were excluded from future tests.

The second test focused on the separating oil, leaving 5 candidates: silicone oil AR 20 (‘AR 20’), 5-cSt silicone oil (‘silicone oil’), mineral oil, tetradecane and hexadecane. Here the final environment was simulated using only two phases, the separating oil and cells in the aqueous phase. In the smaller volume where the surface-to-volume ratio is higher than in the first step, cells prove to be more sensitive as toxic effects were observed sooner (Fig. 3 b). All separating oils gave high viability during a short incubation of 4 h, but after 24 and 48 h some toxicity became apparent. For example, earlier we saw ~80% viability in tetradecane plus Span 80 after a 4-h incubation (Fig. 2 b); here, viability was higher without surfactant, but complete cell death was observed after 24 h (Fig. 3b). For the next step, only fluids giving high viability after the longest incubation period (i.e., mineral oil and 5-cSt silicone oil) were included, even though drug screening would be carried over a shorter period.

In the third test, 5-cSt silicone oil and mineral oil were tested for their suitability. Two 1-μl drops of cell culture media were initially separated by 200 nl oil in HFE-7500. First, a 1% weight-to-weight surfactant in oil was used, followed by serial dilution down to the lowest concentration giving reliable drop merging. As the two oils have different properties, different surfactant concentrations allow merging (e.g., 0.5% in mineral oil, and 0.25% in 5-cSt silicone oil). Next, Jurkats were incubated in a Teflon tube in 2-μl drops engulfed in separating fluid/surfactant (and HFE-7500 as the carrier), and viability measured (Fig. 3 c). Mineral oil + Span 80 proved particularly toxic. 5-cSt silicone oil with all surfactants gave good viability, but Abil EM180 was selected as the surfactant as it maintained drop architecture best during incubation.

As a final step, the effects of 5-cSt silicone oil + 0.25% Abil EM180 in a 3-phase system on levels of 5S rRNA were tested; cells in drops-in-drops behave like controls (Fig. 3 d).

### A proof-of-principle drug screen

**Fig. 4:**
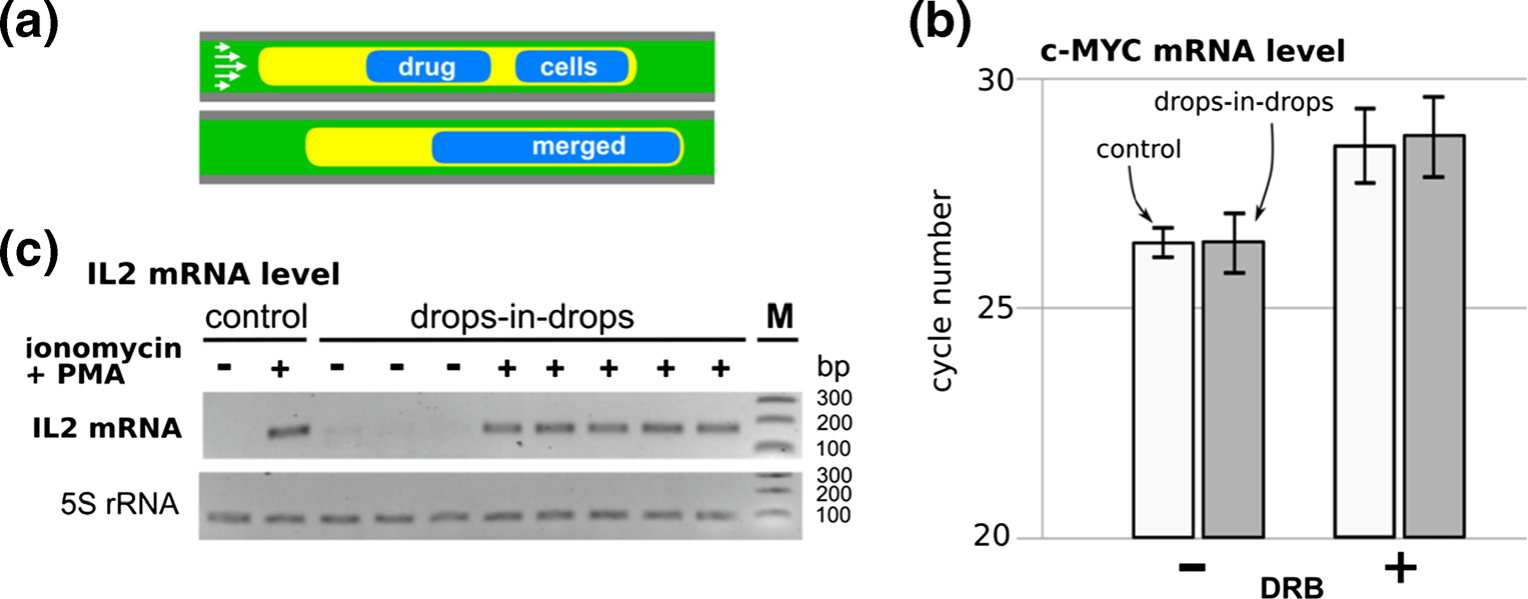
Jurkat cells in drops-in-drops respond to drugs like controls grown conventionally. (**a**) Schematic illustration of the drug screening (HFE-7500 as carrier, 5-cSt silicone oil + 0.25% Abil EM180 as separating oil). (**b**) Effects of DRB on c-MYC mRNA levels (assessed using qRT-PCR). DRB increases cycle number, indicating it reduces mRNA levels; conventional (‘control’) cells behave like those in drops-in-drops. (**c**) Effects of ionomycin + PMA on IL2 mRNA levels (assessed using RT-PCR and gel electrophoresis). 171-bp band indicative of IL2 mRNA is only seen after treatment with ionomycin and PMA; conventional (‘control’) cells behave like those in drops-in-drops. M: 100-, 200-, 300-bp markers. This Figure was redrawn using data from supplementary information in reference (Feuerborn et al. 2015)

Having established which combination of fluids to use (i.e., HFE-7500, 5-cSt silicone oil + 0.25% Abil EM180, medium containing cells), we performed a proof-of-concept drug screen (Feuerborn et al. 2015; supplementary information). DRB (6-dichloro-1-β-D-ribofuranosyl-benzimidazole) is a general transcriptional inhibitor, and – when a drop containing it is merged with another containing Jurkats as in Fig. 4a, levels of c-MYC mRNA (assessed by qRT-PCR) are repressed (in Fig. 4b, more cycles are required, indicative of depression of levels). A drug pair – ionomycin plus phorbol 12-myristate 13-acetate (PMA) – initiate an inflammatory response, and this leads to an increase in levels of interleukin-2 (IL2) mRNA; when a drop containing the pair is merged with another containing cells, levels of IL2 mRNA (assessed using RT-PCR and gel electrophoresis) increase (in Fig. 4c, the band indicative of IL2 mRNA appears). In both cases, cells in drops-in-drops behave like controls grown conventionally (Fig. 4a and b). These experiments showed that HFE-7500 and 5-cSt silicone oil + 0.25% Abil EM180 can deliver both biocompatibility and the interfacial tensions necessary for the merging of drops in a Teflon tube – and so this combination is suitable for drug screening using our assay.

## Conclusion

Different fluids and surfactants commonly used in drop-based microfluidic system have been screened for their biocompatibilities. Genetic analysis showed that cell viability alone was a less reliable indicator of fluid and surfactant biocompatibility. For drops-in-drops applications involving mammalian cells, HFE-7500 as the carrier fluid and 5-cSt silicone oil + 0.25% Abil EM180 as the separating oil showed comparable performance in gene expression tests (4 h incubation time) to the conventional method. Therefore this fluid and surfactant combination is recommended for drug screening. Further works need to be done to assess cell proliferation in the tube for longer time periods using these and/or other fluids. Although here we assume that the incompatibility comes from the fluid and surfactant which are in contact with cell media, there might also be contributions from the tube material (Jiang et al. 2015; Panaro et al. 2004); hence tube biocompatibility and alternative tube materials could also be explored in future works. Finally we suggest that results obtained with many fluid and surfactant combinations and cell-based microfluidics should be interpreted with caution.
